# Traumatic brain injury and the pathways to cerebral tau accumulation

**DOI:** 10.3389/fneur.2023.1239653

**Published:** 2023-08-11

**Authors:** William P. Flavin, Helia Hosseini, Jeffrey W. Ruberti, H. Pirouz Kavehpour, Christopher C. Giza, Mayumi L. Prins

**Affiliations:** ^1^Department of Neurology, David Geffen School of Medicine, UCLA, Los Angeles, CA, United States; ^2^Steve Tisch BrainSPORT Program, Department of Pediatrics and Neurosurgery, David Geffen School of Medicine, UCLA, Los Angeles, CA, United States; ^3^Department of Bioengineering, UCLA, Los Angeles, CA, United States; ^4^Department of Bioengineering, Northeastern University, Boston, MA, United States; ^5^Department of Mechanical and Aerospace Engineering, UCLA, Los Angeles, CA, United States; ^6^Department of Neurosurgery, Brain Injury Research Center, David Geffen School of Medicine, UCLA, Los Angeles, CA, United States

**Keywords:** tau, traumatic brain injury, neurodegeneration, brain pathology, repetitive head injury

## Abstract

Tau is a protein that has received national mainstream recognition for its potential negative impact to the brain. This review succinctly provides information on the structure of tau and its normal physiological functions, including in hibernation and changes throughout the estrus cycle. There are many pathways involved in phosphorylating tau including diabetes, stroke, Alzheimer’s disease (AD), brain injury, aging, and drug use. The common mechanisms for these processes are put into context with changes observed in mild and repetitive mild traumatic brain injury (TBI). The phosphorylation of tau is a part of the progression to pathology, but the ability for tau to aggregate and propagate is also addressed. Summarizing both the functional and dysfunctional roles of tau can help advance our understanding of this complex protein, improve our care for individuals with a history of TBI, and lead to development of therapeutic interventions to prevent or reverse tau-mediated neurodegeneration.

## Introduction

In 1975, while studying the assembly of tubulin into microtubules, the Kirschner laboratory discovered a protein they named “tau,” short for tubulin-associated unit, for its ability to induce tubule formation (1). Then, in 1986, Grundke-Iqbal showed that paired helical filaments, which make up the characteristic neurofibrillary tangles (NFTs) seen in post-mortem brain tissue from Alzheimer’s disease (AD) patients, were composed of tau protein in a hyperphosphorylated state (2, 3). Later, in 1991, Braak and Braak found a characteristic distribution pattern of tau-containing neurofibrillary changes in AD brains (4), thus creating an eponymous staging system which led to an understanding of tau spatiotemporal spread as a causal factor underlying disease progression and severity.

In the past decades since the discovery of a linkage between tau and neurodegenerative disease, there has been intense focus on understanding the mechanisms and risk factors underlying aggregation and propagation of tau pathology in numerous neurodegenerative tauopathies such as AD, chronic traumatic encephalopathy (CTE), frontotemporal dementia (FTD), and progressive supranuclear palsy (PSP), among others. The role of genetic mutations as well as post-translational modifications of the tau protein in the development of neurodegeneration are beginning to be understood, but numerous questions remain unanswered and the goal of therapeutic intervention to prevent aggregation, promote degradation, or halt disease progression remains elusive. One of the most established risk factors for tauopathy is traumatic brain injury (TBI), though despite the link between a history of TBI and the later development of AD or CTE, central questions regarding the mechanistic links are yet unanswered.

This review provides information on the structure of tau, from the gene encoding its six isoforms to the spectrum of possible post-translational modifications, as well as the physiological functions of this protein. In the second part of this review, tau pathology is discussed. We first focus on tau phosphorylation and several pathways that are known to increase this modified form of cellular tau, from diabetes to AD to inflammation and repetitive mild TBI (rmTBI). We then review current knowledge regarding tau pathology following mild and repetitive TBI, including efforts to measure tau as a biomarker for TBI and to quantify tau aggregation *in vivo* using neuroimaging. We discuss pre-clinical research models of TBI-induced tau aggregation and highlight both the benefits and limitations of current models. Finally, we consider tau as a prion-like self-aggregating protein and summarize mechanisms known to underlie spatiotemporal spreading between cells and between more distant brain regions, ultimately leading to disease progression. We have aimed to advance the understanding of the molecular mechanisms involved in tau aggregation and propagation for all neurodegenerative tauopathies, but also to consider TBI as a complex environmental risk factor that can predispose to and drive pathology in unique ways, thus requiring future research to achieve a deeper understanding of this process.

## The tau protein

### Tau gene and protein

**Figure 1:**
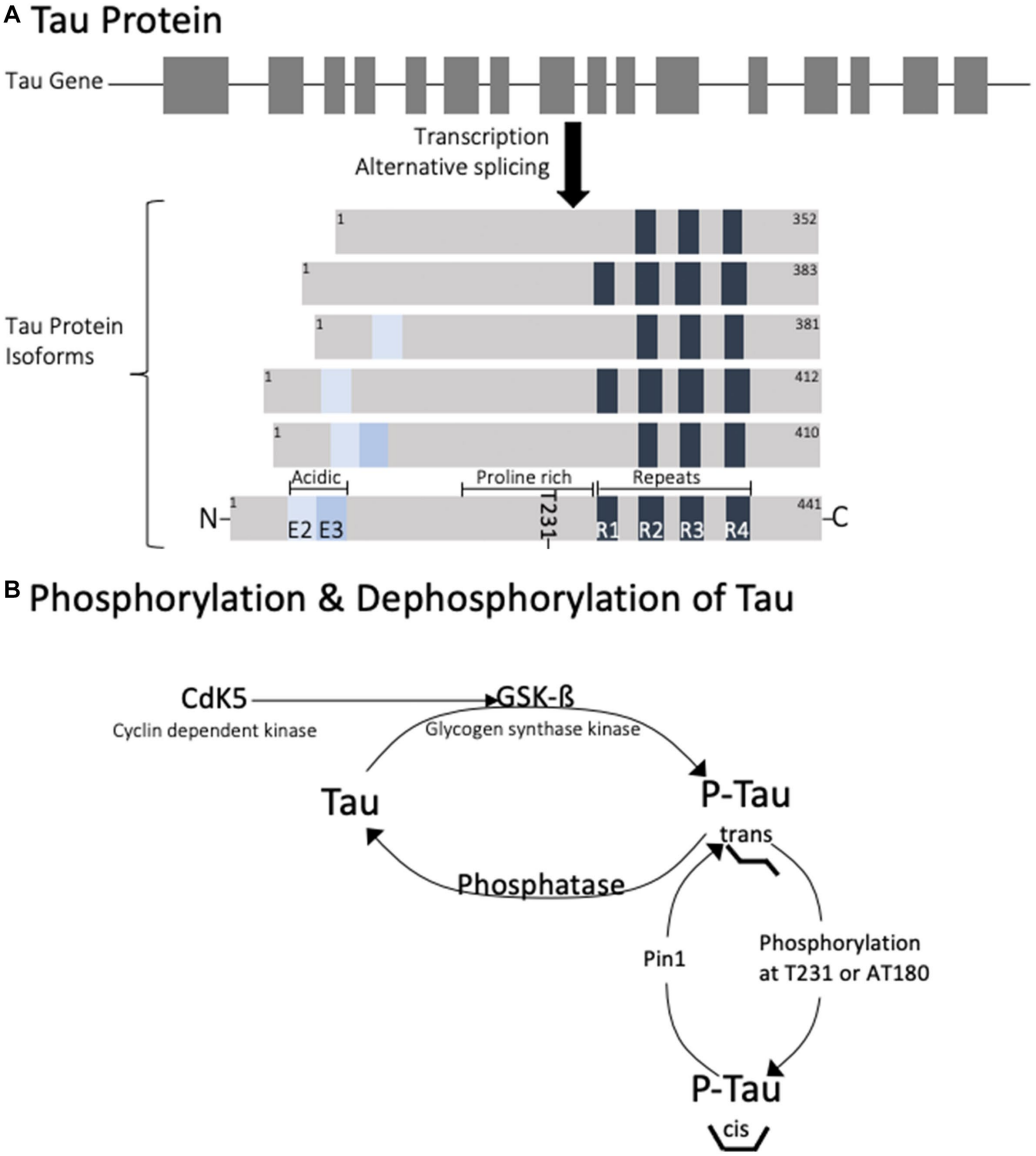
Diagram of the genetic splicing that produces Tau isoforms **(A)** and the enzymes involved in physiological regulation of tau phosphorylation **(B)**. Note that phosphorylation of tau at T231 is responsible for trans to cis conformational change.

Tau is a cellular protein that belongs to the microtubule-associated protein (MAP) family and is predominantly found in neurons, though also present in glial cells. The tau gene is located on the long arm of chromosome 17, with 16 exons spanning over 100 kb (5). In humans, exons 4A, 6, and 8 are not present in mRNA, so the alternative splicing of the remaining exons contributes to the 6 protein isoforms that are generated (6). The proteins range from 352 to 441 amino acids with distinct regions (Figure 1). At the C-terminus, the isoforms differ by the presence of 3–4 repeat regions (3R or 4R) which define a microtubule binding domain. This repeat region is involved in axonal transport, development, and the phosphorylation state of tau. Adjacent to these repeats is a basic proline-rich segment which contributes to microtubule binding. At the N-terminus, there are 0–2 inserts of acidic regions (0 N, 1 N, or 2 N) involved in interaction with other cytoskeletal elements, cytoplasmic organelles, and plasma membranes.

The normal cellular distribution of tau in the brain shows that it is predominantly found in neurons and to a lesser extent in human astrocytes (7). In neurons, tau is present at higher levels in axons than in dendrites (8). This distribution pattern reflects its role in axonal process extension and retraction as well as dendritic spine elongation. Under normal physiologic states the intracellular concentration of tau in a neuron is estimated between 2 and 4 μM (9), (10) with over 99% bound to microtubules (11). Estimations of extracellular concentrations of tau have been obtained in human cerebrospinal fluid (CSF) showing tau around 200 ng/mL (12), (13) and phosphorylated tau at 37.4 ng/mL (13).

### Posttranslational modification

There are numerous posttranslational modifications of tau that affect its function. Details of the functional consequences of tau modifications are presented in several reviews (5, 6, 14). The 40 lysine residues can undergo acetylation, ubiquitylation and SUMO (Small Ubiquitin-like Modifier)-ylation. Acetylation of lysine decreases tau binding to microtubules and leads to its aggregation (15). Lysine ubiquitylation increases tau turnover and degradation (16), while SUMOylation can promote phosphorylation of tau and its aggregation (17). In contrast, methylation competes for lysine residues and decreases tau aggregation (18). N-glycosylation, the addition of a saccharide to the N-terminal asparagine, occurs on hyperphosphorylated tau and contributes to its aggregation (19). O-glycosylation, the addition of a saccharide to a hydroxyl oxygen, is instead thought to maintain tau association with microtubules thereby decreasing its aggregation (20). Nitration of a tyrosine residue can decrease the binding strength of tau to microtubules, increasing potential for tau aggregation (10). Glycation occurs in pathological tau when a carbohydrate is added to lysine, generating advanced glycation end products (AGEs), which further contribute to tau aggregation (21).

Phosphorylation is the most extensively studied post translational modification of tau, playing a role in both normal physiologic functions and pathophysiology. Phosphorylation is an important determinant of tau intracellular localization including the axon, somato-dendritic compartment, plasma membrane, and nucleolar organizing regions (22, 23). Axonal tau plays a phosphorylation-dependent role in axonal process extension and retraction, mediated by various kinases acting on serine, threonine, proline and tyrosine. Tau is most abundant in the cytoplasm and a significant proportion of tau has been found in the membrane in an unphosphorylated state. One study using mice showed that inhibition of casein kinase 1 (CK1) or glycogen synthase kinase 3 (GSK3) leads to a decrease in tau phosphorylation and an increase in its membrane fraction. However, the localization of tau to the membrane where it can interact with membrane associated proteins is dependent on Fyn kinase activation (24). Fyn-tau interaction regulates the NMDA receptor signaling *via* binding to the NR2B subunit involved in the post synaptic dendrite signaling (5). Tau also has a role in maintenance and protection of DNA and RNA in the nucleus (5). In dendrites, tau is responsible for dendritic spine development, elongation and maturation (25). While glycogen synthase kinase (GSK) is responsible for neurite retraction, cyclin dependent kinase (CDK5) is an important regulator in neuronal positioning during development and presumably in synaptogenesis and neurotransmission.

**Figure 2:**
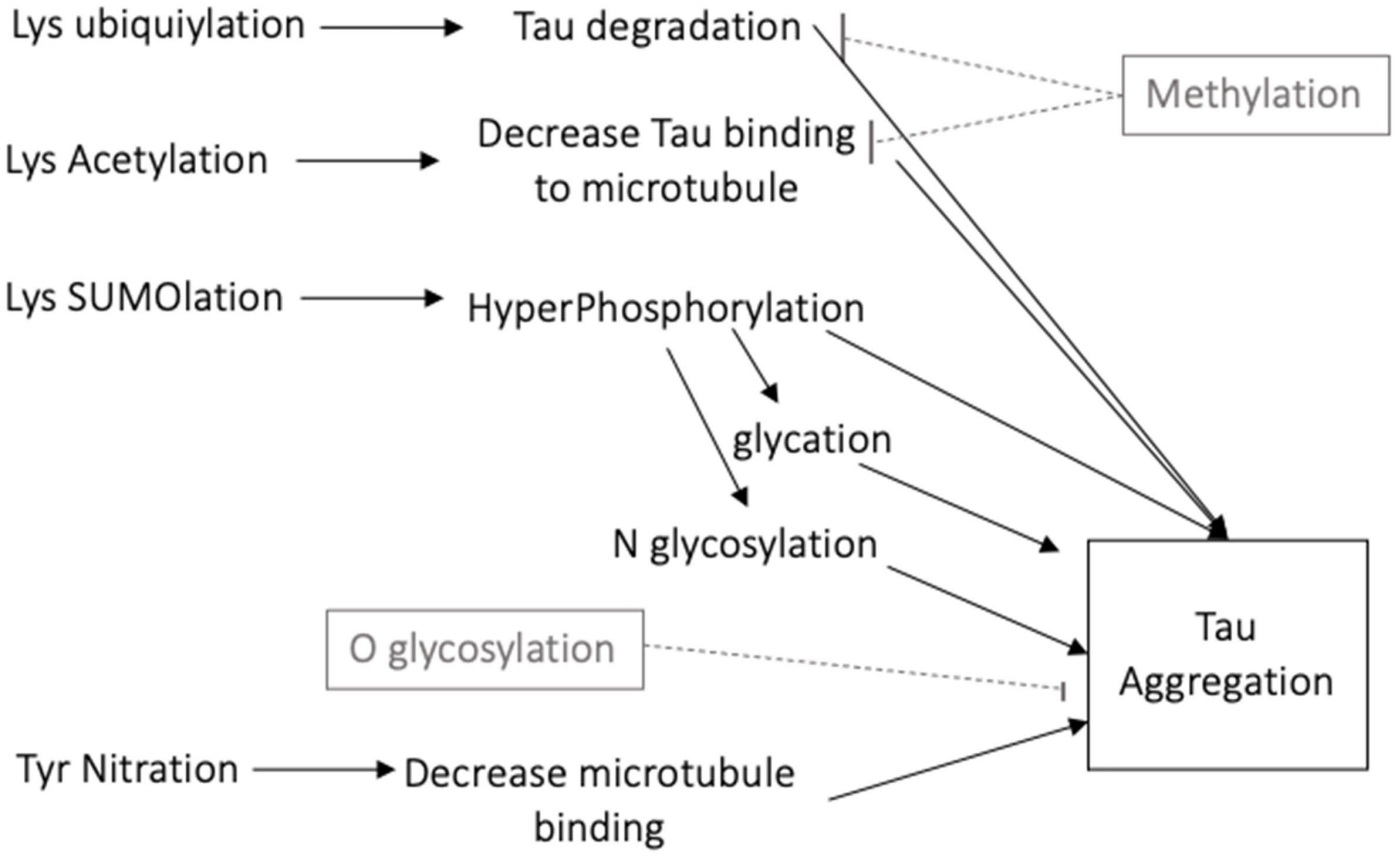
Schematic diagram of the many posttranslational modifications of tau that increase or decrease tau aggregation.

The various cellular functions of tau are regulated through phosphorylation. In the dephosphorylated state tau aggregation is minimal (Figure 2). Phosphorylation of tau is required for physiological functions, but it is tightly regulated. Hyperphosphorylation of tau has been associated with protein aggregation. Normally, 90% of tau exists in the *trans* conformational state which promotes microtubule stability (26). Phosphorylation at Thr231 causes a conformational shift towards the *cis* isomeric orientation, which destabilizes microtubule binding and promotes tau aggregation. *Cis* phosphorylation of tau has been proposed to be a pathological state of the tau protein (27, 28). Pin1 is a prolyl isomerase that dephosphorylates T231 and re-establishes microtubule binding (29). It is currently considered a therapeutic target for diseases caused by tau hyperphosphorylation.

### Tau turnover

A major determinant of a protein’s half-life in the cell is the interplay of its degradation and stabilization signals. The 3R and 4R isoforms of tau have different turnover rates, with 4R having a faster rate relative to 3R isoforms. In human induced pluripotent stem cells (iPSCs), the half-life of tau protein is between 6 to 7 days, and in mature CNS this period is estimated to be 23–24 days (30). The half-life of the protein is determined by the rate of degradation, which occurs through the ubiquitin/protease system (UPS) or the autophagy lysosome pathway (ALP). In the UPS system, tau protein is ubiquitylated and degraded in the lysosome or proteasome (5). The 20S core of proteasomes can be blocked with lactacystin *in vitro*, resulting in decreased degradation of tau (31). Decreased proteasome function may contribute to tau accumulation in both physiologic and pathologic conditions. Other modifications are effective in altering the half-life and function of tau; acetylation by histone acetylases (HATs) increases the stability of tau by acetylating the lysine residues (17). Furthermore, SUMOylation of the lysine 340 residue prevents its ubiquitylation as well as promotes its phosphorylation (17). The mechanisms underpinning targeting of tau to ALP-mediated degradation and its relationship to proteasomal degradation remains incompletely understood.

## Physiologic tau regulation

Tau phosphorylation has been linked to normal physiological conditions including states of synaptic plasticity. One well-characterized and predictable model of plasticity that has been used to examine changes in inter-neuronal connectivity is hibernation. Hibernation is an adaptive behavioral strategy to reduce energy expenditure when environmental conditions are unfavorable. The cyclical changes in metabolism, heart and respiratory rate, and neuronal functions are predictable (32). During torpor, neuronal hippocampal synaptic connections decrease, a process that requires activation of cellular microtubule regulation. One cellular mechanism that can regulate these processes is the phosphorylation of tau. In fact, increased phosphorylation of tau is observed during torpor in cortical brains of arctic ground squirrels (33). Upon arousal from hibernation, tau phosphorylation decreases, synaptic connections are reinnervated and metabolism returns to normal. In the ground squirrel, tau hyperphosphorylation was observed at six sites (S199, T205, S214, S262, S396, and S404), but only three of these are dephosphorylated upon arousal (S199, S262, and S404) (34). This pattern of reversible tau phosphorylation has also been observed in the black bear and Syrian hamster (33), but may differ between cerebral regions and phosphorylation sites.

Evidence from hibernation studies has illuminated the relationship between metabolic substrates and the phosphorylation of tau (35). During torpor, cerebral metabolism switches from glucose to ketone use, a fuel change which is thought to induce tau phosphorylation and morphological change. Injection of non-metabolizable 2-deoxy-d-glucose (2DG) into the rat cortex will effectively starve cells metabolically, forcing them to shift towards other oxidizable energy sources. During this time, there was an increase in ß-APP expression and tau phosphorylation measured by the phospho-tau (Ser202 and Thr205) monoclonal antibody AT8 at 7 h post injection, specifically with an increase in *cis* conformation (36). Reversible tau hyperphosphorylation, primarily in the hippocampus, has also been observed in mice when hypoglycemia was induced by 1–3 days of food deprivation (37). It has been proposed that the decrease in body temperature associated with induced hypoglycemia directly affects kinase and phosphatase activities leading to increased tau phosphorylation (38).

Another physiologic condition associated with significant neuronal plasticity occurs in females during different phases of hormonal changes. In younger pregnant females, there are significant cerebral synaptic changes occurring that require changes in cellular microtubules. Studies have shown that total tau content in the hippocampus decreases during pregnancy, but the proportion of phosphorylated tau increases in rats (39, 40). Female estrogen activates Akt, which inhibits GSK3 thereby inhibiting phosphorylation of tau (41). Later during menopause, decreases in estrogen can reverse the balance between kinases and phosphatases resulting in greater production of p-tau (39). The influence of the estrus cycle and of lifetime hormonal changes on tau regulation and pathology remains incompletely understood.

## Pathways to tau phosphorylation

**Figure 3:**
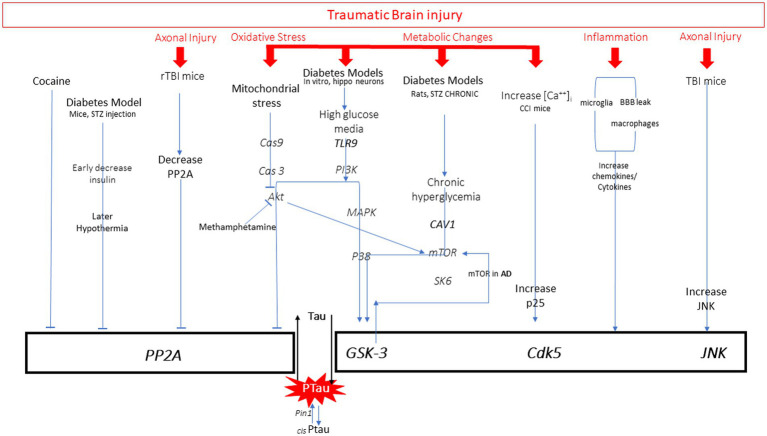
All pathways to tau phosphorylation from numerous diseases and exposures based on literature. It is important to note that those in red are all pathway elements initiated by traumatic brain injury.

There are many pathways leading to pathological increases in tau phosphorylation, including diabetes, stroke, Alzheimer’s disease (AD), brain injury, aging, and drug use. Examination of the mechanisms described by preclinical research models of these diseases reveals common targets for hyperphosphorylation of tau: activation of kinases or inhibition of protein phosphatases. Evidence from various disease models that utilize these pathways will be briefly reviewed (Figure 3). There are several kinases that have been shown to phosphorylate tau at different sites, including glycogen synthase kinase (GSK3ß), cyclin dependent kinase (CDK5), and c-Jun N terminal kinase (JNK) (42). GSK3ß is so far the most commonly activated kinase in pathological tau hyperphosphorylation. This kinase phosphorylates the greatest number of sites on tau. Normally it is involved in cellular proliferation, migration, glucose regulation and cell death. CDK5 activity is highest in neurons, and with the regulator p35 it is involved in brain development (43). When CDK5 complexes with p35 it can phosphorylate tau. JNK is a mitogen activated protein kinase that is involved in cell proliferation, differentiation, development, inflammation and cell death.

### Diabetes/Alzheimer’s disease

Activation of one or more kinases is a common mechanism to increase tau phosphorylation. Preclinical models of diabetes have examined the relationship between diabetes-induced hyperglycemia, hypothermia, and insulin dysfunction on p-tau. Tau hyperphosphorylation can be achieved through several mechanisms that activate kinases GSK3 and CDK5. Cultured hippocampal neurons have been used to study effects of high glucose on p-tau *in vitro*. Sun et al. (44) found increased TLR9 expression in neurons and an increase in tau hyperphosphorylation in the presence of high glucose media. Inhibition of P38/MAPK reversed tau phosphorylation. The GSK3 kinase has also been reported to be activated by hyperglycemia through the decrease in Caveolin-1 (Cav-1), which activates mTOR/SK6 pathway to activate GSK3, thereby increasing tau phosphorylation (45). Many of these pathways involved in diabetes are common to those seen activated in AD. Activation of mTOR has played a prominent role in Aß production and tau phosphorylation through GSK3 activation, which can serve as a feedback loop activating mTOR (46).

Another mechanism that can contribute to hyperphosphorylation of tau is the inhibition of protein phosphatases. This mechanism has been observed under diabetic conditions. Animal models of diabetes can be induced by streptozotocin (STZ) injections which result in decreased circulating insulin and hypothermia. Using this model in mice, it was demonstrated that both decreased insulin and hypothermia can each independently increase phosphorylation of tau (38). Examination of the role of multiple kinases in this process revealed that kinase overactivation is not the primary mechanism, but rather increases in the C-subunit of the PP2A phosphatase enzyme inhibits its function and thereby contributes to the net increase in tau phosphorylation.

### Stress, drugs

Kinases are also influenced by stress, inflammation and drug use to mediate tau phosphorylation. Through corticotropin releasing factor receptor (CRFR) pathways, 30 min of restraint stress in mice can also activate several of the kinases that increase tau phosphorylation (47). Mitochondrial stress with release of cytochrome c can initiate Cas9/3 activation which inhibits Akt and thereby increases activation of GSK3ß (48). This mechanism is also associated with increased p-tau in cell cultures exposed to methamphetamine (49). Rats exposed to cocaine do not show changes in CDK5, but do show inhibition of protein phosphatases, which results in less dephosphorylation of tau and results in p-tau accumulation (50). It is important to note that p-tau is elevated in individuals with drug addictions (heroin), showing greater numbers of regions affected and higher p-tau load than age-matched controls (51). Examination of different drug mechanisms is important given this growing body of evidence.

### Inflammation

Most disease processes initiate pro-inflammatory responses including release of cytokines. These are elevated in diabetes, AD, TBI and other neurodegenerative disorders. Physiological stress, oxidative stress and pro-inflammatory cytokines can activate the JNK pathway (52). Inhibition of JNK in rat neuronal cultures reduced p-tau, suggesting a role for JNK in tau hyperphosphorylation (53). The role of activated microglia has been studied by injecting IL-1ß into the ventricular space in human alpha-1-antichymotrypsin and tau transgene mice (54). In both mouse models, IL-1ß induced phosphorylation of tau that correlated with JNK activity. Induction of the inflammatory response by injection of lipopolysaccharide (LPS) also induces p-tau through the same pathway (55). IL-1ß is the most studied pro-inflammatory molecule in the tau pathway. However, there is mixed evidence that TNFalpha and IL-6 also can play a role through CDK5, but more studies are required. The type of kinase activated has been shown to be dependent on the duration of the immune stimuli and the age of the subject. Chronic LPS delivery to young transgenic AD mice activates CDK5, whereas GSK3ß is the preferred kinase activated in the aged mice (55). It also has been suggested that the activation of the neuroinflammatory responses through LPS may be sufficient to induce changes, but not to sustain persistent tau phosphorylation. This is an important area of research as the exact mechanism of immune-mediated tau phosphorylation is incompletely understood.

### Mild traumatic brain injury and repeat TBI

TBI occurs when the brain moves rapidly inside the skull. This rapid movement causes mechanical deformation of the brain initiating many pathways simultaneously. Initially, the brain movement causes indiscriminate release of neurotransmitters from neurons, which then activate postsynaptic channels allowing large concentrations of ions to move down their concentration gradient. This occurs immediately upon impact, causing an increase in demand for cellular energy as the cells struggle to re-establish ionic equilibrium. Glucose uptake increases early after injury as it is rapidly processed to ATP to fuel the Na^+^/K^+^-ATPase. The phase of hyperglycolysis lasts approximately 6 h post injury, and is followed by a more prolonged state of decreased cerebral glucose metabolism that can last for days-weeks in animal models depending on injury severity and age. Depressed glucose metabolism is often accompanied by decreases in cerebral blood flow, and deficits in motor, cognitive and behavioral modalities have been observed during this time (56). Given the relationship between diabetes, AD-related glucose changes, and tau phosphorylation, it is possible that this represents one mechanism through which rmTBI may also contribute to p-tau accumulation. There are currently no studies examining rmTBI metabolism and tau hyperphosphorylation.

Following mTBI there is an increase in both extracellular K^+^ and intracellular Ca^++^ caused by intense glutamate release. The accumulation of ^45^Ca^++^ after lateral fluid percussion injury is dependent on age and time after injury (57, 58). Immediately after injury, ^45^Ca^++^ accumulated in the cortex in P17, P28, and adult rats and resolved within 4 days. However, in P28 and adult rats there was a second increase at 2–4 days in the thalamus that lasted until 14 days post-injury. The presence of increased calcium can activate calcium-dependent cysteine protease pathways that can contribute to axonal cytoskeletal disruption. Transient increases of intracellular Ca^++^, over 2–5 min, can also lead to an increase in tau phosphorylation in a “dose-dependent” manner, directly correlating with increases in intracellular Ca^++^. This phosphorylation is achieved by GSK3ß activation (59, 60). Increases in intracellular calcium have also been associated with increased tau phosphorylation 24 h after CCI injury in mice. The post-TBI increase in calcium increased conversion of p35 to p25 producing elevated levels of CDK5/p25 complex that are involved in tau phosphorylation (61).

Single and repetitive mild TBI have been shown to produce varying degrees of axonal injury in preclinical models (62, 63) and human TBI (64). Mechanical disruption of the axons can physically disrupt axonal architecture, and increases in calcium can activate calpain-mediated cytoskeletal degradation as well. These processes can disrupt axonal transport and release tau from microtubules. While the exact mechanism remains unknown, one study demonstrated that three fluid percussion impacts in adult rats results in decreased PP2A, which thereby increases tau phosphorylation. Administration of sodium selenate to the animals elevated PP2A and reversed the p-tau accumulation (65). While TBI can induce tau phosphorylation through decreasing phosphatases, other studies have shown activation of kinases as the mechanism. In one study, a single CCI injury in TgAD mice increased tau phosphorylation through JNK but did not change PKA, CDK5 or GSK3ß activity (66). This contrasts with the Yousuf 2016 study, where increases in intracellular calcium were shown to activate CDK5/p25 complex (61). It is unclear why CCI injuries in separate studies show differential kinase activation, but it is clear more research is required to clarify these differences.

TBI also influences tau phosphorylation through the subsequent inflammation occurring after injury. The temporal profile of the inflammatory response after injury is dependent on age, sex, and injury severity. Acute increases in cytokines have been documented after mTBI in human subjects over 1–3 months (67) and in mice over 7–14 days (68). A preclinical study examining mild TBI with weight drop in adult mice showed evidence of blood brain barrier disruption and increases in serum cytokines such as IL-6 at 90 min post injury (69). There are fewer studies addressing the mechanistic link between rTBI, cytokine profiles, and their consequent effect on tau. Madathil and colleagues (70) examined the effects of single and repetitive mild TBI with a closed head concussive injury in adult rats on microglial morphology over time. After a single mTBI, microglia were found presenting both M1 (pro-inflammatory) and M2 (anti-inflammatory) markers at 6 h. While the pro-inflammatory markers returned to baseline 72 h after a single mTBI, the M1 phenotype remained elevated in the rTBI group at this time point, suggesting a persistent activation of microglia with increasing TBI exposure. No studies have addressed the influence of injury interval on the magnitude and duration of pro-inflammatory responses in the setting of repeat TBI.

### Mechanical stretching

TBI is initiated by a mechanical event and initiates numerous biological cascades that compound the physical movement. Biomolecules in solutions exposed to strain can aggregate and change conformation. Recently, tau was studied under extensional flow to determine if strain alone can induce tau aggregation (71). Tau 3R0N (36.7 kDa) was divided into 10 μL droplets and subjected to extensional strain in a modified tensiometer. The diameter of the filament that formed during extension was tracked as a function of time and analyzed for signs of aggregation using tau and two molecules of similar or greater size. Results demonstrated that at high extensional strains, tau protein solution 36.7 kDa and the polyethylene oxide 35 kDa formed aggregates. This is the first study to demonstrate the effects of strain alone on tau protein can cause aggregation. Future studies will need to address how phosphorylated tau behaves and whether specific neuronal microenvironments may create unique strains during injury that contribute to aggregation foci.

## Pathological tau changes: evidence of tau after mild and repetitive traumatic brain injury

### Clinical mild traumatic brain injury and tauopathy observations

Tau has many different but incompletely understood roles in the setting of traumatic brain injury (TBI), particularly mild TBI (mTBI). The largest body of literature comprises neuropathology cases showing evidence of p-tau in the brains of individuals with a history of repetitive head impacts (predominantly American football players, but also other contact sport athletes and military servicemembers). Cases with these autopsy findings have been diagnosed with chronic traumatic encephalopathy (CTE), and consensus neuropathological criteria have been published and updated (72). Research criteria for diagnosis of CTE in living patients have also been published (73), but difficulty remains due to lack of specificity of common symptoms and the potential contribution of ongoing comorbid conditions on neurobehavioral dysfunction in many patients (74). In these settings, most subjects had a prior history of neurological and behavioral changes that preceded death, and an association between repetitive head impacts and neurocognitive impairment or even neurodegeneration has been reported. Tau has also been examined as a fluid biomarker in CSF and blood after mTBI, and newer molecular ligands are permitting *in vivo* PET imaging of potential cerebral tau accumulation in case series (75). However, the challenge remains that cerebral tau neuropathology is also reported in individuals without a history of TBI, and the associations reported in case series are not able to determine whether a causal relationship exists between repeated head impacts and the pathological features described.

### Tau pathology associated with repeated mTBI, but also those with no TBI

Many case studies have demonstrated hyperphosphorylated tau in brain at autopsy in individuals with a history of repeated head impacts, concussions or mTBI (76, 77). CTE has been defined by the pattern of neuropathology showing hyperphosphorylated tau in a perivascular distribution at the depths of cortical sulci (72). These cases are predominantly in professional American football players, but cases have also been reported in other sports (78) and in military service personnel (79). Other case series looking for CTE neuropathology in military personnel found no evidence of neurodegeneration in a cross-sectional study of 18 military service members (80). While cross-sectional studies have proposed a classification system of increasing severity for CTE across stages, neurodegenerative progression has not clearly been demonstrated in prospective longitudinal studies.

Confounding the causal interpretation of repeated head impacts leading to CTE, neuropathology consistent with CTE has also been described in individuals with no prior history of TBI, including epilepsy (81), substance abuse (82), ALS (83), multiple system atrophy (84) and other neurodegenerative diseases (85). These conflicting data raise the possibility that hyperphosphorylated tau CTE neuropathology may not necessarily be progressive nor be specifically associated with repetitive head impacts (86, 87).

### Tau as a biomarker of TBI, mTBI and head impacts

While tau protein may serve as a surrogate measure for axonal injury acutely after TBI, the ability to measure it *in vivo* is limited. In patients with severe TBI, higher levels of tau in the extracellular fluid, measured using microdialysis, correlated with worse clinical outcomes 6 months post-injury (88). Other studies in severe TBI showed acute elevations of total tau in CSF and associations with long-term outcomes (89, 90). However, total or p-tau did not show CSF elevations chronically after more severe TBI (91). In amateur boxers, total tau was elevated in CSF *via* lumbar puncture days after a fight, and normalized within 1–3 months (92). Interestingly, a study of TBI biomarkers (including tau) in amateur soccer players found no significant elevations in CSF sampled within a week of a heading training session (93). Some studies of concussion reported elevations of serum tau compared to non-concussed controls, but methodological differences in assays may confound interpretation (89, 94). Ultrasensitive detection of total tau (t-tau) using the Simoa assay kits (Quanterix) has shown increased plasma t-tau levels 1 h after concussion in professional ice hockey players, compared to pre-season levels, and these levels were predictive of time to return-to-play (89). Other studies of mTBI suggest that cleaved tau is a poor predictor of long-term outcome or development of post-concussion syndrome following mTBI (95, 96). In 105 emergency department patients, t-tau levels drawn within 24 h of mTBI were correlated with ordinal outcome Glasgow Outcome Score-Extended (GOSE) but not Rivermead Post-Concussion Symptom Questionnaire (RPCSQ) scores (97). In military personnel undergoing training to breach or force entry into dangerous environments, tau levels in blood 1 h after exposure to blast overpressure did not significantly differ from pre-exposure levels (98). Other chronic military studies showed no significant differences in serum tau between controls and those with a history of prior TBI, but did report significant correlations between tau and symptoms (99). The association of CSF and serum tau levels with mTBI and their relationship to long-term outcomes remains unclear.

### Tau neuroimaging in those with a history of repeated mTBI

More recently, studies have begun using radioisotope ligands to attempt to demonstrate tau deposition in living patients (100–102). There have been reports of clinico-pathological correlates between antemortem tau-PET and subsequent CTE tau neuropathology on autopsy (103, 104). Using post-mortem brain tissue samples of pathologically confirmed CTE, autoradiography with tau PET tracer AV-1451 (flortaucipir) did not detect robust binding. This suggests that tau PET tracers may have different binding patterns for different tau conformations in different tauopathies (105). Better characterizing these methods and further longitudinal studies in patients with suspected CTE will be important to more clearly determine progression over time.

In summary, tau has been implicated in both the acute response to repetitive mTBI (rmTBI) and in the setting of chronic neurobehavioral impairment seen in some patients with a history of prior repeat head impact exposure and concussions. While neuropathological consensus criteria for a diagnosis of CTE have been proposed, clinical criteria for this diagnosis proposed for research purposes are nonspecific and overlap with many treatable comorbidities (73, 106), limiting the present utility of these criteria for patients. Further work is necessary to distinguish neuropathology associated with CTE from other conditions that have shown similar findings. Lastly, better prospective longitudinal delineation of neurobehavioral impairments in conjunction with worsening p-tau neuropathology is needed to confirm whether CTE is a progressive illness; advances in *in vivo* detection using fluid and imaging molecular biomarkers show promise in these critical investigations.

### Preclinical mild TBI and tauopathy research

This section will review the existing literature specifically addressing mTBI and rmTBI in animal models with tau outcomes. Many rmTBI models have recently emerged in an effort to address current sports injury concerns. However, in modeling a concussive injury several important criteria must be met. First, the model must demonstrate that the single impact is histologically and behaviorally consistent with clinical mild concussive injuries, for example not causing bleeding, skull fracture, transient behavioral deficits, or any gross pathology. Second, the model must involve a rmTBI protocol that is relevant to the clinical scenario. Lastly, appropriate control groups must be included and relevant time points after injury must be selected to examine acute versus chronic effects. These experimental specifics have been summarized in Table 1. Previous research has established that age at injury influences outcome (57, 107–109) therefore the studies will be grouped into adolescent/young adult, adults, and aged rodents.

**Table 1:** Experimental Models of Repeat Mild TBI and Tau.

Young	Subjects	Injury model	RTBI	PID	Tau finding
Gao (110)	6 weeks rat, male	CCI, open skull, in stereotaxic, lateral	1X, rTBI (4x, 24 h int)	1–30 days	Increase pTau 1-30 days with peak at 14 days. Suggest transient expression
Mouzon (112)	10–12 week hTau mice, male	CCI, closed head, midline	1X, rTBI (5x, 48 h int)	1 day, 12 months	Transient increase pTau expression, not chronic
Wright (111)	30 days, rat, Both sex	Pneumatic barrel, lateral	1X, rTBI (3x, 4 days int)	22 days	Increase pTau in rTBI group for both sexes
Adult	Subjects	Injury model	RTBI	PID	Tau finding
Laurer (113)	Mice, male	CCI, open skull, in stereotaxic, lateral	1X, rTBI (2X, 24 h int)	1, 3, 7, 56 days	No changes in pTau with IHC
Hoshino (114)	Rats, Male	FPI, lateral	1x	2, 4, 6 months	Semiquantitative Increase AT8 pTau, IHC cortex at 2, 4, 6 months
Rubenstein (115)	TghTau/PS1 Mice, Both sexes	CCI, closed head in stereotaxic, midline	rTBI (4x @ 72 h int)	1, 10 days, 2, 4, 6, 8, 10, 12 months	In cortex, total tau and pTau increased between 1–10 days and remained elevated to 12 months
Petraglia (129)	Mice, Male	CCI, on helmet no anesthesia	1x, rTBI (42x with 6x per day @ 2 h int for 7 days)	7 days, 1 month, 6 months	Increase pTau with single that recovers by 6 months, but remains elevated in rTBI in cortex not hippocampus
Zhang (117)	Rats, Male	Pendulum, frontal midline	rTBI (4X, 12 h int)	14 days	Decrease pTau by Western blot
Kane (118)	Mice, sex not indicated	Weight drop, foil *10% mortality with 5x	1x, rTBI (5x, 10X @1 day int)	30 days	Increase pTau at 30 days after 5X rTBI
Tadepalli (116)	Rat, Male	Weight drop, exposed skull, disc, midline	1x, rTBI (5x @24 h int), rapTBI (5x @5 m int)	1 day, 56 days	1 day IHC-no change in Tau in brainstem 8 weeks serum ELISA no change in pTau
Haar (119)	Rats, Male	CHIMERA, Frontal injury	rTBI (5x @ 2 week int)	77 days	Faint pTau IHC in mesolimbic structures
McAteer (122)	Rat, Male	Weight drop, exposed skull, disc, midline	1x, rTBI (3x at 5 days int)	1 day, 84 days	Increase AT180 with 3x at 1day, but magnitude of increase decreased at 12 weeks post injury
Luo (120)	GFAP luciferase mice, Male	CCI, closed head, rubber tip	1x, rTBI (2x, 3x, 5x @24 h int)	90 days	IHC show interspersed pTau labelled cells but not quantified
Ojo (121)	hTau Mice, both sex	CCI, closed skull, in stereotaxic, midline	24-32x (2x @72-96 h int. weekly for 3–4 months)	180 days	Mild increase in pTau (t231-RZ3) in cortex. No change in pTau with CP13 and PHF1 antibodies
Aged	Subjects	Injury model	RTBI	PID	Tau finding
Hawkins (123)	Rat 400–500 g, Male	FPI, midline	1x, severity unclear	4, 24 h, 14 days	Increase in tau oligomers at 4 and 24 h in cortex and hippocampus
Ojo (124)	hTau Mice, 18 months, both sex	CCI, closed head, in stereotaxic	1x, rTBI (5x @48 h in for 9 days)	21 days	At 18 months sham show tau pathology. rTBI increase pTau in cortex and hippo
Ojo (130)	PSI/APP Mice, 16 months both sex	CCI, closed head, in stereotaxic	rTBI (12x in 1 month)	30 days	Increase CP13 in rTBI PSAPP mice. No change in WT or other tau antibodies
Yoshiyama (125)	T44 Mice, 12 months, both sex	CCI, closed head, rubber tip	rTBI (16x, 4x per day, 1 week int for 4 weeks)- no shams	9 months	1 mouse showed neurofibrillary tangles and cerebral atrophy

CCI (controlled cortical impact), FPI (fluid percussion injury), CHIMERA (closed-head impact model of engineered rotational acceleration), PID (post-injury date).

Consistent with TBI research as a whole, few studies have addressed changes in tau phosphorylation after rmTBI in the adolescent age group. Gao and colleagues examined p-tau within the first month after single and repetitive mTBI and found that p-tau increases during the time period, peaking around 14 days post injury (110). This was observed in both males and females (111, 112). In a rmTBI model using human tau mice, Mouzon and colleagues observed increased p-tau expression at 1d post injury, but not at the 12 months chronic time point (112). Despite differences in the locations and injury specifics, all the studies suggest transient increase in p-tau expression when rmTBI occurs during adolescence.

While a greater number of studies have addressed changes in p-tau after rmTBI in the adult animal models, the findings are varied. There are a number of variables that contribute to the lack of consensus, including wild type versus transgenic animals as well as different injury models, number of injuries, injury intervals, time after injury, and types of p-tau analysis and antibodies used. Among the studies utilizing multiple time points, one study found no changes in tau using immunohistochemistry (113) and three studies demonstrated initial increase in total phosphorylated tau within the first 7–10 days that endured at 6–12 months after rmTBI (114, 115). It is important to note the differences in subjects, injury model, and number of injuries and intervals between these studies. Those studies examining a single acute (< 30 days) timepoint also showed variability; a weight drop injury showed no tau histology changes in brainstem at 1d (116), a frontal pendulum rmTBI showed decreased pTau at 14 days (117), and a weight drop in mice showed increased p-tau at 30 days (118). The remaining studies examining a single chronic time point showed some increase in p-tau but they were not robust (119–121), and one study showed p-tau even decreased compared to 1 day (122). There is a growing need to examine the time course of tau phosphorylation after injury to determine how number of impacts, impact interval, and time contribute to eventual pathology and dysfunction. A single injury may induce more transient plasticity-related changes in tau, whereas repeated injuries with short intervals may result in pathological tau accumulation. Achieving consensus using wild type or transgenic models remains difficult to achieve with so many variations in experimental designs and multiple regional analyses.

Only a handful of studies have addressed TBI-induced tau phosphorylation in the aged animal, and three of the four were conducted in transgenic animals. Increased tau oligomers were observed at 4 and 24 h post injury in the cortex and hippocampus in aged male rats following fluid percussion injury (121, 123). Tau pathology was quite variable in transgenic mice with increases observed with specific antibodies (121, 124) at 21–30 days post injury. RmTBI in a TgT44 mouse model only found 1 animal with neurofibrillary tangles 9 months after injury (125). Quantification of tau pathology in the aged rodent presents several difficulties, as aged sham animals will also develop significant pathology, especially in transgenic animals, and show age related decline in behaviors. Regardless of age, these rmTBI animal models examining tau phosphorylation reveal the difficulties of establishing consensus without investing in a more comprehensive study that takes sex, age, time course and tau quantification into account.

## Aggregation and propagation of p-tau

### Tau as a prion-like amyloid protein

Misfolding of tau leads to its pathological aggregation from a natively unstructured soluble monomer to a higher-order insoluble hyperphosphorylated multimer in a beta-sheet fibrillar conformation (126, 127). It is this aberrant aggregation and accumulation of tau as seen in neuropil threads (NTs) or neurofibrillary tangles (NFTs) that are the pathological hallmark and defining feature of diverse tauopathies such as Alzheimer’s disease (AD), chronic traumatic encephalopathy (CTE), Pick’s disease (PiD), progressive supranuclear palsy (PSP), argyrophilic grain disease, and corticobasal degeneration (CBD), among others (127). Filaments composed of all 6 tau isoforms make up NFTs in AD and CTE, whereas tangles in PiD include predominantly 3R isoforms and tangles in PSP, AGD, and CBD are composed of mostly 4R isoforms (128).

Though the mechanism by which native tau becomes misfolded and begins to aggregate remains uncertain, missense mutations (P301S and P301L) in the microtubule-binding domain of tau that cause familial frontotemporal dementia have implicated destabilization of microtubule binding in promoting aggregation (131, 132). Physiological phosphorylation of tau occurs as part of its cellular function as discussed previously, but NFTs are known to be hyperphosphorylated compared with normal tau, and phosphorylation has been shown to decrease microtubule binding affinity and promote fibrillization (127, 131). This phosphorylation-driven fibrillization may occur through a liquid–liquid phase separation of tau microtubule-binding repeats that induces aggregation through molecular crowding and electrostatic forces in liquid droplets (133, 134). Binding of tau to microtubules is also thought to be disrupted due to axonal injury during TBI, where microtubule disassembly causes tau dissociation and predisposes it to aggregation, a process hypothesized to underlie CTE pathogenesis (135). We have shown that mechanical stress in the form of extensional strain causes tau to aggregate in solution (71), and others have observed phosphorylated tauopathy induced by concussive or severe TBI in mouse models (79, 136–138). Though the example of familial FTD shows tau aggregation alone is sufficient to cause neurodegeneration, other disease-related amyloid proteins are known to interact with tau and promote its aggregation; interactions with amyloid-beta (Aβ) (139–141) or alpha-synuclein (α-syn) (142, 143) are best studied. Finally, tau aggregation has been triggered by inflammatory stimuli such as inflammasome activation in microglial cells (144, 145), though involvement of additional glial cell populations such as astrocytes or oligodendrocytes may also contribute (146, 147). Once aggregated, since the microtubule-binding region comprises the core of tau filaments, tau aggregation has been shown to be both a toxic gain-and loss-of-function (148–150), as the pathological assembly and physiological function of tau appear to be mutually exclusive.

**Figure 4:**
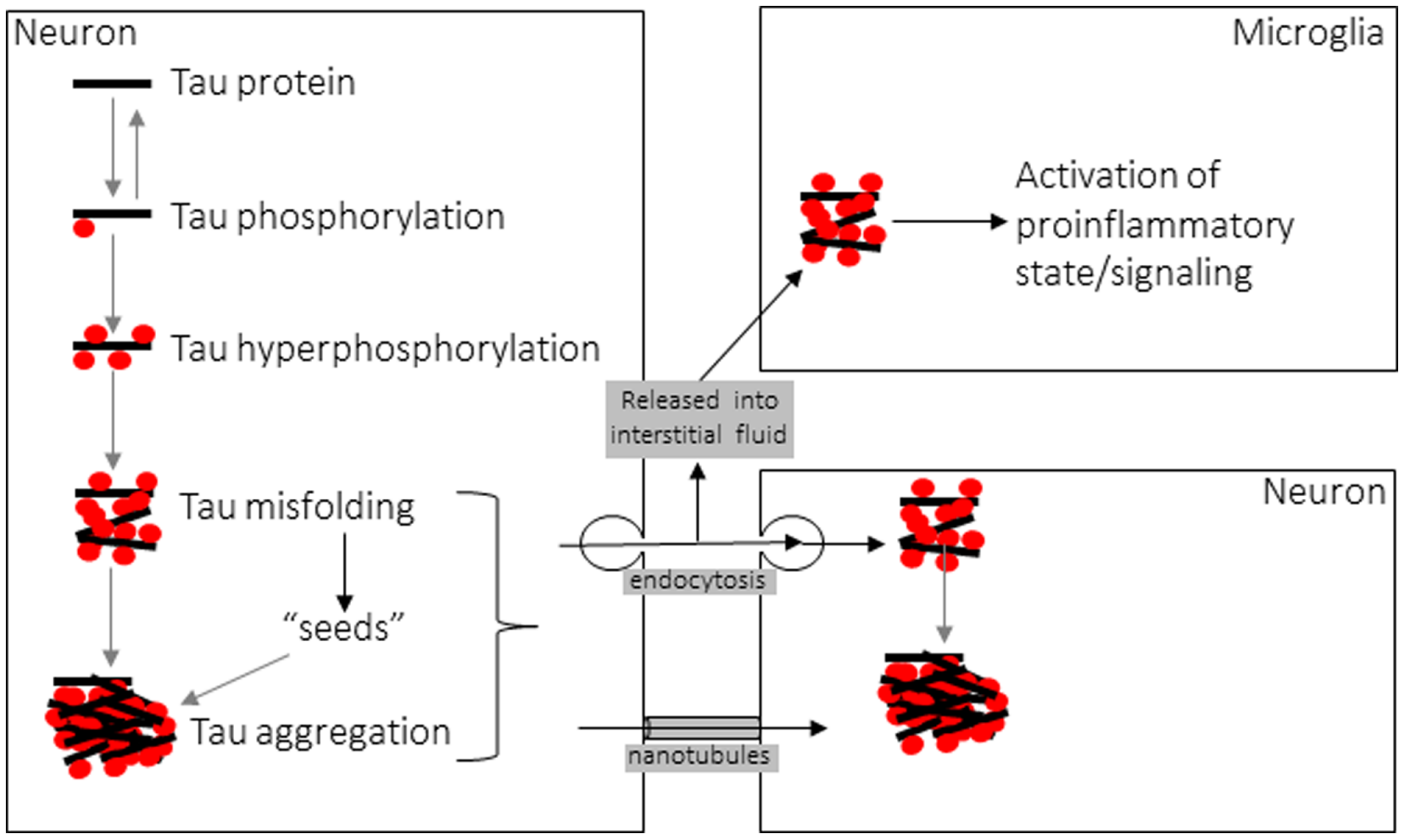
Diagram of Tau aggregation and propagation between cells.

New insight into the etiology and progression of tauopathies, and numerous other neurodegenerative proteinopathies, stems from the 1982 discovery by Prusiner that a protein alone is the etiological agent of transmissible spongiform encephalopathies such as Creutzfeldt-Jacob disease (CJD). He termed these proteins prions, for proteinaceous infectious particles (151), and in the years since this discovery numerous other proteins, with tau among them, have been shown to behave in a similar fashion to CJD prions at the cellular and biochemical level (128, 135, 152), with the notable exception being that no other disease-related amyloid protein has been shown to be infectious or transmissible between hosts as in CJD (153). In addition to the aforementioned conversion from a physiological, soluble form to a disease-associated, insoluble amyloid form, a hallmark of prions and “prion-like” proteins such as tau is that they amplify their pathological misfolded state through templated recruitment of additional soluble counterparts, triggering further misfolding when naïve monomers contact the misfolded “seed” (Figure 4) (154, 155). Numerous cell culture studies and both transgenic and wild-type animal models have demonstrated the ability of tau fibrils to induce this seed-driven templated amplification of further aggregation, recapitulating Prusiner’s protein-only hypothesis by the induction of NFT insoluble aggregates following cellular uptake of tau fibrils (156–158). Seeding of further aggregation can be inhibited by suppressing the expression of soluble tau, in which case tau aggregates can then be cleared by the autophagy-lysosome pathway and the ubiquitin-proteasome system, though these systems are unable to clear large aggregates (159). Intracellular tau inclusions are known to be dynamic, with fibrillar components undergoing frequent fragmentation and growth facilitating pathological propagation by additional seeding events (159). Fluorescence resonance energy transfer (FRET)-based methods of detecting tau seeds have shown that seeds precede development of NFTs and conventional phosphorylated tau histopathology (160, 161). There is considerable debate regarding which specific composition of tau multimer comprises the minimum seed-competent species, with data from tau mutant transgenic mice suggesting small filamentous species > 10 monomeric components (162), whereas other data demonstrating even tau monomer, derived from sonication of larger tau fibrils, encodes structural information to induce seeding (163).

Another hallmark of prions is structural heterogeneity, or misfolding into multiple self-replicating conformations that define unique “strains,” where each conformer produces distinct disease phenotypes that underlie the clinical diversity of prion diseases caused by aggregates of the same protein. Tau aggregation also exhibits this structural polymorphism, resulting in unique strain conformations specified by the templating process and influenced by numerous factors such as isoform composition and post-translational modifications (114, 164). Indeed, seed-competent tau monomers are capable of reconstituting multiple distinct strains owing to different stable monomeric conformations (165). When tau fibrils from different tauopathies are isolated from human brain, the structural polymorphism that defines unique strains has been identified using cryo-electron microscopy, with novel folds observed in AD, CTE, PiD, and CBD (166, 167). Strain conformation is consequential because it imparts unique properties to individual polymorphs. Tau strains exhibit varying levels of potency in their seeding of further aggregation, as strains purified from AD brain exhibited increased seeding potency compared with synthetic tau fibrils (168, 169). Furthermore, strain conformation imparts a unique neuroanatomical and cellular tropism resulting in distinct patterns of neurotoxicity in different tauopathies (170, 171). A specific strain conformation and its associated pathological phenotype of affected cell types and brain regions can be faithfully reproduced when tau strains are re-introduced into naïve cells or successively inoculated into mice (155, 172). These findings highlight how templated amplification of protein misfolding and strain-specific differences in clinicopathologic phenotype define tau as a prion-like amyloid protein, and have given rise to a deeper understanding of the mechanisms underpinning disease progression in tauopathies. While these prion-like mechanisms have been better studied in the context of AD and other primary tauopathies, the overlap with these mechanisms and CTE pathogenesis remains incompletely understood. While McKee and others have hypothesized a similar prion-like behavior of tau in CTE as in other tauopathies (135), a deeper mechanistic understanding is still needed to elucidate whether tau can adopt these prion-like roles following the complex environmental insult of TBI.

### Propagation of tau between brain regions

Another prion-like feature of tau pathology is the transmission of aggregated tau from cell-to-cell and between brain regions. Braak described a tau staging system in AD with six stages that distinguish a progressive stereotypical distribution pattern for tau pathology that correlates well with disease severity (4, 173). This characteristic sequential progression of pathological tau in AD is thought to begin in the locus ceruleus and transentorhinal cortex and then spread spatiotemporally through olfactory bulb, hippocampus, and entorhinal regions, and finally neocortical regions such as basal temporal, temporal, insular, and frontal cortices. A similar 4-stage system has been used to describe the ordered and predictable progression of tau pathology in CTE, which co-occurs with axonal disruption and death (72). More recent positron emission tomography (PET) studies using tau-specific ligands in AD and CTE have supported the respective tau staging systems previously established by histological examination of post-mortem brain tissue (174, 175). Though differing tauopathies affect unique circuits, progression staging schemes have also been described for PSP (176), AGD (177), and PiD (178). These findings support the idea that the expansion of misfolded tau pathology through cell-to-cell propagation contributes to differing clinical phenotypes and may be a driver of clinical disease progression.

Intercellular propagation of assembled tau has been seen in numerous experimental models, where expressed cellular tau adopts characteristics of the pathological exogenous tau seeds and this protein misfolding cascade spreads to neighboring cells and interconnected brain regions. Various forms of aggregated tau have been used as seeds for intracerebral injection in animal models, including pre-formed fibrils (PFFs) derived from *in vitro* assembly of mutant or WT human or mouse tau, brain extracts from transgenic mutant P301S or P301L human tau overexpressing mice, or pathologic tau purified from brains of patients with AD, CBD, AGD, or PSP. Additionally, tau pathology has been initiated *via* regional overexpression in transgenic animals or transduction of specific brain regions with lentiviral vectors in non-transgenic animals (179, 180). In keeping with the behavior of distinct strains, when seeds derived from tauopathy patients are injected into brains of transgenic mutant or WT tau overexpressing mice or non-transgenic mice, they caused templated amplification of misfolding and cell-to-cell propagation of endogenous tau aggregates with a cell-type and spatiotemporal phenotype that recapitulated the hallmark disease lesions of the extract source (169, 171, 172). Cell-type specificity in tau aggregate transmission is perhaps best illustrated in the case of tau extracts derived from postmortem AD, PSP, and CBD brains injected into non-transgenic mice, where all extracts induced neuronal tau inclusions but only PSP and CBD tau strains were capable of inducing astroglial and oligodendroglial inclusions, a pattern which recapitulates authentic human neuropathology (170). Interestingly, and as further evidence that structural differences endow strains with a unique bioactivity, oligodendroglial tau aggregates induced by PSP or CBD tau strains can propagate to downstream cells along white-matter tracts even when neuronal tau is knocked down, whereas astrocytic tau aggregates do not have this ability (147). Within affected circuits, whether tau aggregates were injected or triggered by local overexpression, synaptic connections appear to enhance tau propagation (179, 180), although transmission can also occur *via* non-synaptic mechanisms such as along glymphatic or CSF pathways (135). Strain-specific differences in spreading are also observed, with various tau strains exhibiting different rates of propagation (181, 182). This prion-like intercellular spreading of misfolded tau represents a mechanism for the amplification and dissemination of tau pathology throughout the brain, and thus understanding the mechanisms underlying cellular uptake and release of tau aggregates are of utmost importance.

### Propagation of tau between cells

In the intercellular propagation life cycle of fibrillar tau, many similarities to prions are again noted when examining mechanisms of cellular entry and egress (Figure 4). Fibrillar assemblies of aggregated tau are predominantly taken up into recipient cells *via* clathrin-mediated or fluid-phase bulk endocytosis (158, 183), occurring after interaction at the plasma membrane between tau aggregates and heparan sulfate proteoglycans (HSPGs) (184). Recent work has also established a role for low-density lipoprotein receptor-related protein 1 (LRP1) in facilitating tau uptake *via* endocytosis and subsequent propagation to downstream neurons (185). Expectedly, both aggregate conformation and size dictate entry *via* endocytosis, as low-molecular weight aggregates or short fibrils, but not monomers, long fibrils, or long filaments, were capable of cellular entry *via* this mechanism (186). Once contained within the endocytic vesicle, fibrillar tau assemblies are known to induce vesicle rupture as a mechanism of escape from the vesicular compartment to access the cytosol, where templated amplification of further tau aggregation can precede (187). Exogenous tau seeds come into direct contact with intracellular expressed tau as seen by FRET following endocytosis, triggering misfolding (154). In response to this damaging mechanism of cellular invasion, danger receptors such as galectins 3 and 8 detect damaged vesicles and activate autophagy by recruitment of cargo receptors to degrade the burden of amyloid proteins and vesicular debris (187). When seeds are taken up into microglial cells they activate the NLRP3 inflammasome, triggering pro-inflammatory caspase-1 activity and IL-1beta release as well as exacerbating intracellular tau seeding of further aggregation (145, 188). Considering that increasing the degradative burden on proteostatic pathways may predispose these signaling processes and trafficking events to become dysfunctional or overwhelmed, the intracellular consequences of tau aggregate cellular entry may promote further propagation by increasing cellular stress, since lysosomal or autophagic dysfunction are causes of cellular stress known to trigger tau secretion from affected cells (189, 190).

Regarding the process by which tau is released from cells, monomeric tau is constitutively secreted, even from healthy cells, into the interstitial fluid (ISF) and cerebrospinal fluid (CSF) for poorly understood reasons (191, 192), although neuronal activity and calcium influx stimulate this release and blocking pre-synaptic vesicle release inhibits it (193). Secretion of tau occurs through a non-classical pathway that is prevented by low temperature but not blocked by inhibitors of the conventional secretory pathway (194). Because of this secretory mechanism, the resulting extracellular tau is in a membrane-free form, illustrating how antibodies directed against tau can reduce insoluble tau aggregates and inhibit seeding and propagation (154). Tau is detected in CSF and the level correlates with the burden of hyperphosphorylated tau and NFT pathology (195), though ISF levels of monomeric tau are higher and reciprocally decrease as spontaneous or seed-induced aggregation consumes soluble tau into insoluble inclusions (191). Tau is also known to be released from affected cells in a membrane-enclosed form, either in ectosomes or microvesicles formed from outward budding of the plasma membrane or in exosomes released when endosome-derived multivesicular bodies that contain internal vesicles fuse with the plasma membrane (180, 190). Lastly, tau aggregates have been shown to be transmitted between cells *via* tunneling nanotubes (TNTs), transient filamentous-actin-containing membranous conduits between the cytoplasm of interconnected cells, in a manner similar to prions (196, 197). This mode of intercellular propagation does not require an extracellular phase. Interestingly, exogenous tau is also known to promote the formation of TNT connections that can then facilitate its transmission (196, 197). Understanding the molecular mechanisms involved in the cell-to-cell life cycle of fibrillar tau will allow future work to explore novel therapeutic interventions to counteract or prevent these pathologic processes.

## Conclusion

Significant progress has been made to understand the role of tau in neurodegenerative proteinopathies, though tremendous opportunities remain to uncover unique molecular mechanisms that drive individual diseases, and how cellular, genetic, and environmental factors interface to produce distinct pathology in these conditions. Disease-modifying therapeutic approaches that leverage these discoveries would have the potential to benefit millions of patients in the future as our population ages. While the bulk of current research on tau is focused on toxicity questions such as elucidating the causes of *de novo* tau aggregation, as well as understanding mechanisms of seeding further aggregation, release and uptake in neuronal cells, studying the fibrillar structure of tau aggregates isolated from post-mortem brain may also allow insight into differences between individual tauopathies and facilitate improved diagnostics such fluid biomarkers or as tracer ligands for neuroimaging. Additional study is greatly needed to understand the influence of TBI on the development of tau aggregation, with consideration for the degree of TBI severity as well as if TBI occurs in a repetitive manner over a longer period of exposure. We are now beginning to appreciate the wide degree of structural heterogeneity that exists for fibrillar tau in distinct tauopathies, as well as the numerous cellular factors that may influence the adoption of unique misfolded states. This complexity highlights the need for rigorous investigation into strong risk factors such as TBI to determine how this environmental insult to the brain can predispose to chronic neurodegenerative sequelae. Although central questions still remain open, advances in research techniques and model systems along with an increasing focus on patients with a history of prior TBI will allow the research community to meet the challenge of developing future treatments to halt or even prevent tau-related neurodegeneration.

## Author contributions

WPF contributed to writing the aggregation and propagation of tau section, and formulated the final draft. HH contributed to the writing of the original first draft. JWR contributed to the mechanical stretching & tau regulation section along with HPK and CCG contributed to the repeat TBI and clinical history of mild TBI and tau sections. MLP initiated this paper, wrote significant portions and designed the images. All authors listed have made a substantial, direct, and intellectual contribution to the work and approved it for publication.

## Funding

UCLA Brain Injury Research Center, UCLA Steve Tisch BrainSPORT Program, NS110757, NS104311. WPF was supported by NINDS 5R25NS065723.

## Conflict of interest

The authors declare that the research was conducted in the absence of any commercial or financial relationships that could be construed as a potential conflict of interest.

## Publisher’s note

All claims expressed in this article are solely those of the authors and do not necessarily represent those of their affiliated organizations, or those of the publisher, the editors and the reviewers. Any product that may be evaluated in this article, or claim that may be made by its manufacturer, is not guaranteed or endorsed by the publisher.
